# Biological Activities and Chemical Contents of Edible *Hohenbuehelia petaloides* (Bull.) Schulzer

**DOI:** 10.1021/acsomega.4c02369

**Published:** 2024-11-05

**Authors:** Cansu Korkmaz, Hatice Güneş, Meltem Taş Küçükaydın, Selçuk Küçükaydın, Mehmet Emin Duru

**Affiliations:** † Department of Biology, Faculty of Science, Muğla Sıtkı Koçman University, 48000 Muğla, Turkey; ‡ Department of Chemistry, Faculty of Science, Muğla Sıtkı Koçman University, 48000 Muğla, Turkey; § Department of Medical Services and Techniques, Köyceğiz Vocational School of Health Services, Muğla Sıtkı Koçman University, 48800 Köyceğiz/Muğla, Turkey

## Abstract

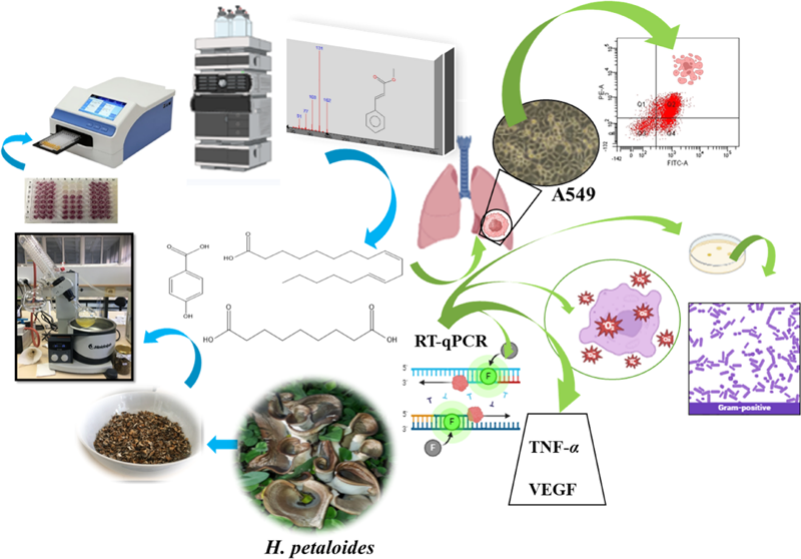

Mushrooms are a good diet with high protein and polyunsaturated fatty acid contents in health, food, and industry from past to present. Mushrooms have attracted a lot of attention in terms of the bioavailability of natural products. *Hohenbuehelia petaloides*, a member of the *Pleuroteceae* family, is an edible wood fungus that grows naturally on the trunks of old and decayed trees. In this study, the cytotoxic activities of hexane, methanol, and water extracts of *H. petaloides* against various cancer cell lines A549, MCF-7, PC-3, and HT-29 were investigated with the 3-(4,5-dimethylthiazol-2-yl)-2,5-dipenyltetrazolium bromide (MTT) assay. In addition, the apoptotic, inflammatory, angiogenic, and antimicrobial effects of the extracts were examined by flow cytometry, real-time quantitative polymerase chain reaction (RT-qPCR), enzyme-linked immunosorbent assay (ELISA), and well diffusion assays, respectively. Moreover, the antioxidant activity and phenolic and lipid components of *H. petaloides* were determined. The hexane extract showed the highest cytotoxic activity (IC_50_ = 26.48 ± 0.02 μg/mL) against A549 cells, while water and methanol extracts exhibited the highest cytotoxicity (IC_50_ = 83.18 ± 0.05 μg/mL and IC_50_ = 90.95 ± 0.05 μg/mL, respectively) against PC-3 cells. The hexane extract killed A549 cells via apoptosis. The methanol extract, at the IC_50_ level, was the most effective in decreasing both tumor necrosis factor-α (TNF-α) and vascular endothelial growth factor (VEGF) release. In antioxidant activity tests performed with 5 different methods, the methanol extract had higher antioxidant activity than the others, followed by 2,2-diphenyl-1-picrylhydrazyl (DPPH) free radical (IC_50_ = 82.61 ± 0.90 μg/mL) and 2,2-azino-bis-3-ethylbenzothiazoline-6-sulfonic acid (ABTS) cation radical removal (IC_50_ = 55.20 ± 0.65 μg/mL) and CUPRAC-reducing power (IC_50_ = 76.41 ± 0.73 μg/mL). Among the extracts studied, the hexane extract showed antimicrobial activity against *Bacillus cereus*, *Staphylococcus aureus*, *Bacillus subtilis*, and *Micrococcus luteus* with different inhibition zones. The major lipid components of *H. petaloides* analyzed by gas chromatography (GC) and gas chromatography–mass spectrometry (GC/MS) were elaidic acid (38.22%), palmitic acid (30.59%), stearic acid (13.21%), linoleic acid (4.35%), and azelaic acid (4.29%). The phenolic compounds determined by the high-performance liquid chromatography with photodiode-array detection (HPLC-DAD) system were *p*-hydroxybenzoic acid (7.42 μg/g extract), cinnamic acid (6.83 μg/g extract), gallic acid (5.36 μg/g extract), and protocatechuic acid (1.83 μg/g extract). The results showed that *H. petaloides* has the potential to be a natural source for the development of novel anticancer and antimicrobial agents as well as a beneficial food supplement for the prevention of cancer.

## Introduction

Currently, multidisciplinary research studies focus on the purification, structural characterization, chemical modification, and biological evaluation of new potential natural anticancer products obtained from different organisms. In order to minimize the side effects of conventional treatment methods and to develop new preclinical targeted combination strategies, there is a need to discover new natural products with anticancer properties and to elucidate their mechanisms.^1,2^ According to the cancer statistics reports, four leading cancers are lung, colon/colorectal, breast and prostate.^3^ Siegel et al. reported that lung, colorectal, and pancreatic cancers account for most deaths so far.^2^ Prostate cancer (29%), lung, and colorectal cancer account for 48% of all cases in men. In women, breast cancer (32%), lung cancer, and colorectal cancer accounted for 51% of all new diagnoses. Approximately 340 people die each day from lung cancer nearly 2.5 times more than the number of people who die from colon/colorectal cancer, which ranks second in cancer deaths.^2^ Murugesan et al. reported that the most serious cancers start in the lungs and intestines of the human body.^4^ Many chemotherapy drugs have resulted in serious side effects.^4^ In recent years, interest in fungi as raw materials in the production of cancer drugs has been increasing.

Macrofungi are a preferred diet because of traditional history due to their rich protein content, low-fat content, and high polyunsaturated fat content. In addition, mushrooms such as *Lentinula edodes*, *Pleurotus citrinopileatus*, *Ganoderma lucidum*, *Schizophillum commune*, and *Fomesfomentarius* have been used for centuries in East Asia, especially in Japan, China, and Korea, as a traditional medicine for various diseases. They are considered as natural resources because of their low toxicity and high specificity for activating the body’s immune system. Moreover, due to their various bioactive compounds (phenolic contents, polysaccharides, proteins, fatty acids, lectins, and ergosterols), edible macrofungi are consumed as a functional food and have attracted the attention of scientists due to their various biological activities such as immunomodulatory, anti-inflammatory, anticancer, antitumor, antioxidant, and antiviral.^5−10^

Essential fatty acids, such as linoleic acid, participate in the formation of high-density lipoprotein (HDL), which transports fat from the blood to the liver, where it is metabolized and reduces the risk of cardiovascular disease.^11^ As reviewed by Sande et al., the percentages and types of fatty acids differ according to the mushroom species and the country from where they were collected, possibly due to climate and soil characteristics.^11^ Linoleic, oleic, palmitic, and stearic acids are the major lipid compounds detected in some mushroom species (*Leucopaxillus gentianeus*, *Armillaria tabescens*, *Suillus granulatus*, *Pleurotus eryngii*, and *Polyporus squamosus*).^12−14^ In addition, the presence of different fatty acids has been reported in different macrofungi species. For example, myristic acid in *Coprinus truncorum* and lauric acid in *C. truncorum* by Karaman et al.; suberic acid in *Trametes bicolor* and cinnamic acid in *T. bicolor* and *Trametes versicolor*, pentadecanoic acids in *Trametes pubescens*, *Trametes suaveolens*, and *T. versicolor* by Çayan et al.; estragole in *Polyporus arcularius* by Fessner et al.; lauric acid in *Cerrena unicolor*, *Inonotus rheades*, *Leptoporus mollis*, and azelaic acid (AZA) in *C. unicolor* by Çayan.^14−17^

Various biological activities of phenolic compounds (antioxidant, antiallergic, anti-inflammatory, antidiabetic, antimicrobial, antipathogenic, antiviral, and antithrombotic properties) have been reported to have a protective role against cancer, cardiovascular diseases, osteoporosis, diabetes mellitus, and neurodegenerative diseases.^5,18,19^ Mizuno and Minato et al. have reported that mushrooms are distinguished as important food containing polysaccharides possessing potent anti-inflammatory and immunomodulating properties.^10^

The genus *Hohenbuehelia*, a member of the *Pleuroteceae* family, grows on dead branches, rotten wood, stumps, bark, or herbaceous stems.^20^*Hohenbuehelia petaloides* (Bull.) Schulzer is an edible mushroom and is naturally distributed in Southeastern Mexico and Anatolia.^21−23^ When the literature is examined, investigations on *H. petaloides* are mostly limited to taxonomic studies; however, the biological activities and chemical content of *H. petaloides* have not been clarified so far. Therefore, the aim of the present study was to investigate the cytotoxic activities of *H. petaloides* extracts against lung (A549), breast (MCF-7), prostate (PC-3), and colon (HT-29) cancer cells, which are the leading causes of cancer-related deaths. In addition, the apoptotic, anti-inflammatory, angiogenic, and antimicrobial activities of the *H. petaloides* extracts were analyzed. Moreover, antioxidant activity, phenolic constituents, and lipid components of *H. petaloides* were determined.

## Materials and Methods

### Mushroom Material

**Figure 1:**
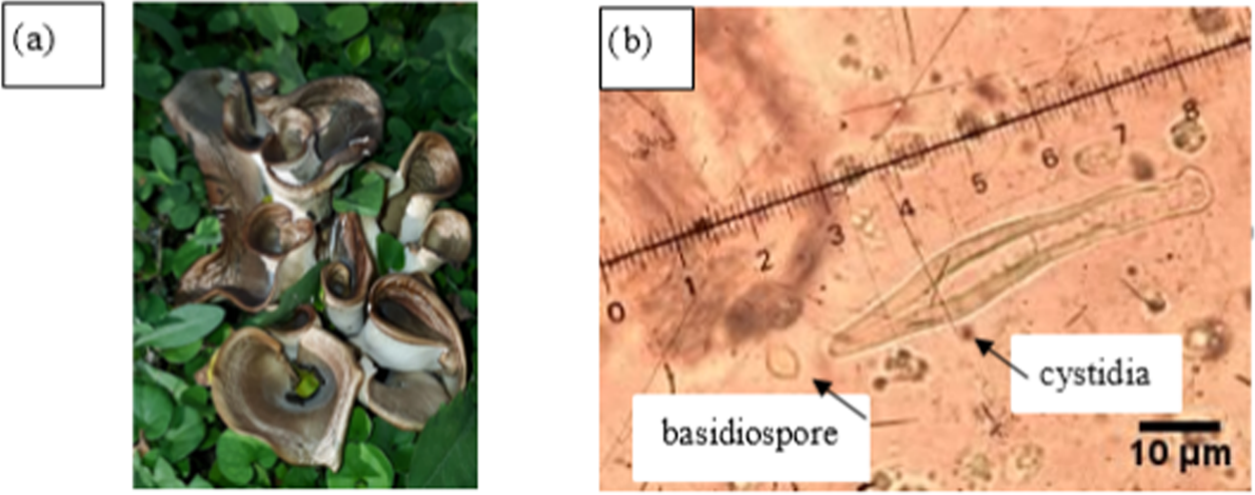
Macroscopic (a) and microscopic (b) views of *H. petaloides*.

In this study, the edible species *H. petaloides* (Bull.) Schulzer was freshly collected from *Dichondra pepens* L. (mouse ear grass) and the woody tree remains in a mixed forest of *Pinus brutia* L., *Pinus pinea* L., and *Phoenix theophrasti* Greuter trees at 36,765 latitude, 27,759 longitude, and 45,124 m altitude between August 2019 and November 2020 in Datça Peninsula, an ecotourism area in the Mediterranean phytogeographic region in Turkey. The fungarium material was deposited in the Natural Products Laboratory of Mula Sıtkı Koçman University under code CK010. The macroscopic and microscopic views of *H. petaloides* species are shown in Figure 1.

### Extraction of Mushroom

Dried mushrooms (300 g) were extracted with hexane and methanol under room conditions (24 h × 3 times), taking into account the direction from the nonpolar to the polar system in the solvent system. The solvent was removed using a rotary evaporator. The remaining material was then infused in hot water (at about 85 °C) for 1 day to prepare for water extraction, and the solvent was removed using a lyophilizer. All extracts were stored at +4 °C.

### Determination of the Chemical Content of *H. petaloides*

#### Determination of Lipid Components

The derivatization process, called methylation, was used in order to determine the lipid components of the *H. petaloides* hexane extract.^24^ About 100 mg of *H. petaloides* hexane extract was weighed. The organic solvent was removed under reduced pressure using a rotary evaporator to obtain methyl esters. Qualitative and quantitative analyses of the methyl derivatives of the lipid fraction of *H. petaloides* were carried out by gas chromatography (GC-FID) and gas chromatography–mass spectrometry (GC–MS), as reported by Küçükaydın and Duru.^24^

### Gas Chromatography (GC) and Gas Chromatography–Mass Spectrometry (GC–MS) Analysis

GC analysis of the methyl derivatives of the lipid fraction of *H. petaloides* was performed using a Shimadzu GC-17 AAF, V3, 230 V series gas chromatograph (Japan) coupled with a flame ionization detector (FID) and a Rxi-5Sil MS fused silica capillary nonpolar column (30 m × 0.25 id., film thickness: 0.25 μm). The injector and detector temperatures were 250 and 270 °C, respectively; the carrier gas was He at a flow rate of 1.4 mL/min; the sample volume was 0.4 μL; and the split ratio was 50:1. The initial oven temperature was held at 100 °C for 5 min, then increased up to 238 °C at 3 °C/min increments, and held at this temperature for 9 min. The relative percentages of compounds were calculated by using the GC Solution computer program.^24^

GC–MS analysis of the methyl derivatives of the lipid fraction of *H. petaloides* was performed using a Varian Saturn 2100T instrument coupled with an ion trap mass spectrometer (MS) and a Rxi-5Sil MS fused silica nonpolar capillary column (30 m × 0.25 mm ID, film thickness 0.25 μm). An electron ionization system with an ionization energy of 70 eV was used for GC–MS detection. Helium (15 psi) was used as the carrier gas at a flow rate of 1.4 mL/min. The injector and MS transfer line temperatures were set to 250 and 200 °C, respectively. The oven temperature was held at 100 °C for 5 min, increased up to 238 °C at 3 °C/min increments, and held at this temperature for 9 min. 0.2 μL was injected manually in the split mode. The split ratio was 50:1. Electron impact mass spectrometry (EI-MS) was performed at an ionization energy of 70 eV. The mass ranged from *m*/*z* 28 to 650 amu.

The components were identified by co-injection with standards (where possible), computer matching with the Wiley, TRLIB, and NIST08 libraries, combined GC retention indices calculated using a homologous series of C_7_–C_30_ alkanes (Supelco), and comparison of fragmentation patterns reported in the literature. Quantification of each of the individual constituents of the hexane extract was based on the internal normalization of the components.

### Determination of Phenolic Constituents

For the determination of phenolic profiles of *H. petaloides* by the high-performance liquid chromatography with photodiode-array detection (HPLC-DAD) system, the mushroom extract was dissolved in the appropriate solvent system (methanol extract) according to the method of Çayan et al.^16^ The same conditions as those used for the reference compounds were applied by injection into an HPLC-DAD system (Shimadzu 20 AT Series HPLC, Japan). Separation was achieved on an Intertsil ODS-3 reverse-phase C_18_ column (5 μm, 250 mm × 4.6 mm i.d), thermostated at 40 °C. The flow rate of the gradient solvent system was 1.0 mL/min. Detection was carried out with a photodiode array detector (PDA) using 280 nm as the preferred wavelength. The sample volume was 20 μL. The mobile phases used were (A) 0.5% acetic acid in water and (B) 0.5% acetic acid in methanol. The elution gradient was as follows: 0–10% B (0–0.01 min); 10–20% B (0.01–5 min); 20–30% B (5–15 min); 30–50% (15–25 min); 50–65% (25–30 min); 65–75% (30–40 min); and 75–90% (40–50 min). The phenolic compounds were characterized according to their retention times, and the UV data were compared with those of the commercial standards. Three parallel analyses were performed. For quantitative analysis of phenolic compounds, calibration curves were obtained by injecting known concentrations of different standard compounds. The HPLC chromatograms of the reference compounds, extracts, and analytical parameters of the identified compounds are provided in the Supporting Information.

### Cell Lines and Culture Conditions

**Figure 2:**
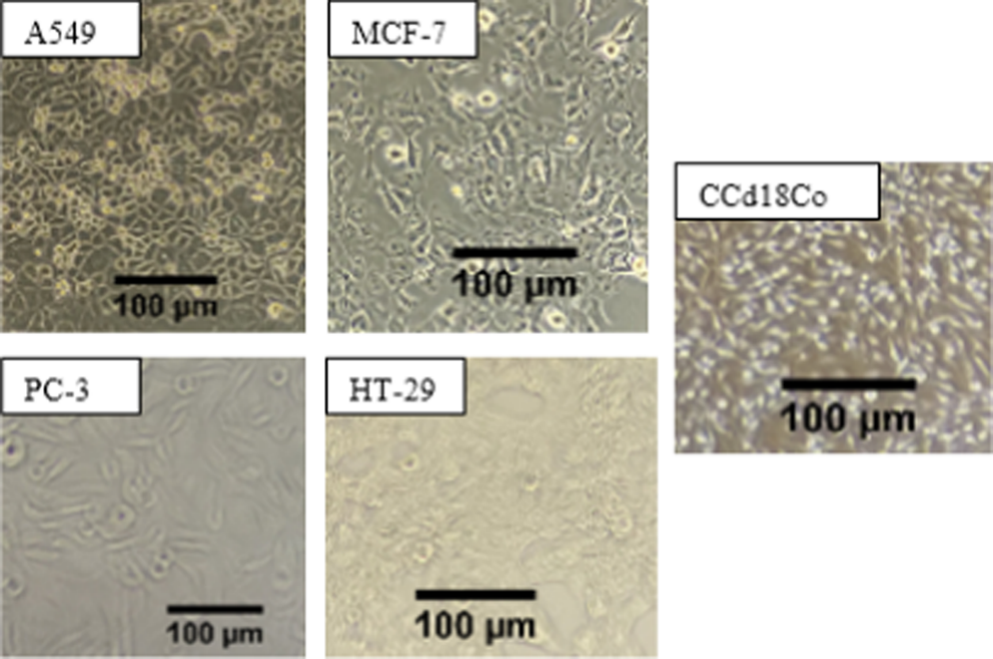
Microscopic views of the lung (A549), breast (MCF-7), prostate (PC-3), and colon (HT-29) cancer cell lines, and healthy human (CCD18Co) cell lines (magnified at x20).

Human lung (A549), breast (MCF-7), prostate (PC-3), and colon (HT-29) cancer cell lines and human healthy fibroblast (CCD18Co) cell lines (obtained from Cell Culture Laboratory, Faculty of Science, Mugla Sıtkı Kocman University) were maintained in RPMI 1640 medium, supplemented with stable l-glutamine, 10% heat-inactivated fetal bovine serum (FBS), penicillin (100 U/mL), and streptomycin sulfate (100 mg/mL) (Biochrom, Germany). All cell lines were incubated in a humidified atmosphere of 5% CO_2_ and 95% air at 37 °C. After the cells were grown to 80% saturation, they were washed with phosphate-buffered saline (PBS). The adherent cells were separated from the surface by trypsinization. Microscopic views of lung (A549), breast (MCF-7), prostate (PC-3), colon (HT-29) cancer cell lines, and healthy human (CCD18Co) cell lines are shown in Figure 2.

### *In Vitro* Cytotoxicity Assay

The cytotoxic effects of hexane, methanol, and water extracts of *H. petaloides* on A549, MCF-7, PC-3, and HT-29 cancer cell lines and healthy (CCD18Co) cell lines were determined using the 3-(4,5-dimethylthiazol-2-yl)-2,5-dipenyltetrazolium bromide (MTT) method.^25^ Cells were seeded in triplicate in 96-well microplates (Greiner) at 1× 10^4^ cells/well and incubated for 24 h before the addition of extracts. The hexane extract was dissolved in 10% dimethylsulfoxide (DMSO), and the methanol and water extracts were dissolved in water. The cells were treated with the extracts (0–0.8 mg/mL) for 72 h. After that, 10 μL of MTT (5 mg/mL) was added into the wells and incubated for 4 h. The medium was discarded, and the formazan crystals were dissolved by adding 100 μL of DMSO. The absorbance was measured at 540 nm with a spectrophotometer (Thermo Scientific, Multiskan FC). Untreated cells served as controls. Cells treated with cisplatin or doxorubicin were used as positive controls. The half-maximal inhibitory concentration (IC_50_) of the extracts was determined using the AAT Bioquest.^26^ The percentage of cytotoxicity was calculated in accordance with the following formula.^27^
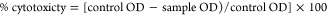


### Apoptosis Assay

#### Investigation of Apoptosis by Flow Cytometry

The annexin V-fluorescein isothiocyanate (FITC)/propidium iodide (PI) co-staining assay was performed according to the manufacturer’s Annexin V-FITC Apoptosis Detection Kit (eBioscience) protocol. Briefly, A549 cells were seeded in 6-well plates at a density of 5 × 10^5^ cells/well. The cells were treated at IC_50_, IC_50_ 1/2, and 2 × IC_50_ concentrations of hexane extracts for 24 h at 37 °C. The cells were trypsinized, washed, and resuspended in a binding buffer. After adding 5 μL of annexin V-FITC and 10 μL of PI (20 μg/mL) to each cell suspension, the cells were incubated in the dark for 15 min. After adding the binding buffer (500 μL), 1× 10^4^ cells of each group were analyzed by flow cytometry (BD FACSCanto A, BD Biosciences) using BD FACSDiva software v6.13.

#### Investigation of Apoptosis by Real-Time Quantitative Polymerase Chain Reaction (qPCR) Analysis

Cells (1 × 10^6^ cells/mL) were treated with different concentrations of the hexane extract of *H. petaloides* for 24 h. RNA extraction was performed using the Nucleospin RNA Plus kit (Macherey Nagel). Two micrograms (2 μg) of total RNA were reverse-transcribed, and first-strand cDNA synthesis was carried out based on the procedure described in the HperScript First Strand Synthesis Kit (GeneAll). The expression profile of the apoptotic gene (*caspase-3*) at the mRNA level was investigated using real-time quantitative PCR (RT-qPCR). RT-qPCR was performed according to the procedure described in the kit A.B.T.TM 2× qPCR SYBR-Green Master Mix (Wizbio Solutions). A real-time PCR machine (LightCycler 480 Roche) was used to amplify the gene sequence for 40 cycles. The thermal cycling conditions were as follows: initial denaturation at 95 °C for 10 min, denaturation at 95 °C for 15 s, and annealing at 60 °C for 1 min. The primer sequences used are given in Table 1.

**Table 1:** Primer Sequences for Real-Time PCR

gene name	primer	primer sequences
β-*aktin*	forward	5′-CCAACCGCGAGAAGATGA-3′
*caspase-3*	reverse	5′-CCAGAGGCGTACAGGGATAG-3′
	reverse	5′-TTGAGGAGTCTCACCCAACC-3′
	forward	5′-TGAAGCTACCTCAAACTTCC-3′

### Quantitative Detection of Human Tumor Necrosis Factor-α (TNF-α) by Enzyme-Linked Immunosorbent Assay (ELISA)

In order to examine the effects of *H. petaloides* extracts (hexane, methanol, and water) on inflammation, 2 × 10^5^ cells/well of A549 cells were cultured in 6-well plates and incubated for 1 h. The cells were treated with 200 μg of each extract along with 10 μg/mL lipopolysaccharide (LPS) for 6 h or left untreated to serve as a control. The supernatants were collected, and 100 μL of each supernatant was tested for inflammatory cytokine tumor necrosis factor (TNF-α) production by ELISA, according to the manufacturer’s instructions of the human ELISA kit (Bioassay Technology Laboratory). The color intensity formed in proportion to the amount of cytokine in the supernatant was determined at a wavelength of 450 nm using a spectrophotometer (Thermo Scientific, Multiskan FC). The amount of TNF-α in the supernatants was calculated using the calibration curve of the standard cytokine.

### Quantitative detection of Human Vascular Endothelial Growth Factor (VEGF) by Enzyme-Linked Immunosorbent Assay

The effect of each mushroom extract on the secretion of vascular endothelial growth factor (VEGF) in the lung cancer cell line A549 was investigated. The cells were cultured in 6-well plates at a density of 2 × 10^5^ cells/well and incubated for 1 h. Then, they were treated with 200 μg of each extract along with 10 μg/mL LPS for 6 h or left untreated as a control. The concentration of VEGF in the cell culture supernatant was determined by ELISA, as described in the manufacturer’s protocol (VEGF Elisa kit, Bioassay Technology Laboratory). The absorbance of each well was measured at 450 nm using a microplate reader. The amount of VEGF in the supernatant was calculated using the calibration curve of the standard.

### Antioxidant Activity

Five different antioxidant activity assays were carried out for hexane, methanol, and water extracts: total antioxidant activity (β-carotene decoloration method), 2,2-diphenyl-1-picrylhydrazyl (DPPH) free radical scavenging activity, 2,2-azino-bis-3-ethylbenzothiazoline-6-sulfonic acid (ABTS) cation radical scavenging activity, Cu(II)-reducing activity (CUPRAC), and metal-binding activity. The assays were performed according to the method described by Taş et al.^28^ BHA, α-tocopherol, and ethylenediaminetetra-acetic Acid (EDTA) standards were used. A graph of the inhibition percentage (%) versus the concentration (μg/mL) was used to calculate the IC_50_ values of the extracts.

### Antimicrobial Activity

The antimicrobial effects of the mushroom extracts were investigated according to the agar well diffusion method described by Balouiri et al.^29^ Vancomycin (35 mg/well) was used as a positive control. Twenty-five milliliters of MHB (Mueller–Hinton broth) containing soft agar (0.7%) was mixed with 50 μL of indicator bacteria or fungi at a density of 0.5 McFarland, poured onto a Petri dish, and allowed to solidify. The samples were incubated at 37 °C for 15 min to completely remove moisture. All extracts were filtered (0.45 μm) and sterilized. Hexane extract (2 mg/well), methanol, or water extract (9 mg/well) were added to the wells and then left at 4 °C for 22 h for diffusion. The next day, the Petri dishes were incubated for 24 h according to the temperature of the indicator bacteria, and zone formation was evaluated.

### Statistical Analysis

Data are expressed as the mean ± standard deviation (SD) of triplicate independent experiments, and analyzed via two-way analysis of variance (ANOVA), followed by Tukey’s multiple comparison using EXCEL PRO 2019-QI Macros, and GraphPad Prism version 8 Software. *p* < 0.05, *p* < 0.01, *p* < 0.001, and *p* < 0.0001 were considered to be statistically significant.

## Results and Discussion

### Determination of the Chemical Content of *H. petaloides*

#### Determination of the Lipid Components

The lipid components of *H. petaloides* are shown in Table 2. Elaidic acid (38.22%), palmitic acid (30.59%), stearic acid (13.21%), linoleic acid (4.35%), azelaic acid (4.29%), and cinnamic acid (2.99%) were the major components (Table 2); and pentanoic acid (0.88%), suberic acid (0.80%), azelaaldehydic acid (0.77%), anethol (0.48%), myristic acid (0.28%), estragol (0.16%), nonanoic acid (0.21%), hydocinnamyl alcohol (0.20%), α-isosafrol (0.26%), ethyl 7-oxooctanoate (0.26%), and ethyl 7-oxooctanoate (0.26%) were the minor compounds. The levels of each lipid component changed from one mushroom species to another. For example, in a previous study, linoleic, oleic, stearic, and palmitic acids were reported as the major fatty acids in the mushroom species *Chondrostereum purpureum*, *Coprinus comatus*, *Gloeophyllum odoratum*, *Daedalea quercina*, *Gloeophyllum trabeum*, *Gloeophyllum sepiarium*, *Hydnum repandum*, *Omphalotus olearius*, *S. commune*, *Phellinus igniarius*, *Phaeolus schweinitzii*, *T. pubescens*, *Inonotus radiatus*, *T. versicolor*, *Macrolepiota procera*, *T. bicolor*, and *T. suaveolens*.^14^ It is known that stearic acid consumption reduces the risk of breast, cervical, prostate, and colon cancers, and inhibits these cancer cells. It was reported that stearic acid lowers cholesterol in coronary heart cardiovascular diseases, decreases the risk of cancer and disease in diabetic patients, protects cortical neurons from oxidative stress by increasing antioxidant enzymes, and reduces the risk of various cancers (prostate, breast, and colorectal).^30^ Işık et al. reported that linoleic acid, which is a polyunsaturated fatty acid, cannot be produced in the human body. Therefore, it is an important essential fatty acid.^31^ Linoleic acid is necessary for human basal metabolism and has many beneficial effects.^32,33^ Palmitic acid is a common saturated long-chain fatty acid known to exhibit anti-inflammatory and metabolic regulatory effects, and antimicrobial and antitumor activities in various tumor types.^34,35^ In a study conducted by Zhu et al., palmitic acid was found to inhibit prostate cancer cell proliferation and metastasis by suppressing the phosphotidyldinositol 3-kinase/threonine protein kinase PI3K/Akt pathway.^34^ Elaidic acid has been reported to inhibit human neuroblastoma (SH-SY5Y) cell growth and induce apoptosis by increasing oxidative stress.^36^

**Table 2:** Lipid Components of *H. petaloides*

compound name	RI	*H. petaloides*	*H. petaloides* %
2-hexenol	849	35,234	0.18
benzaldehyde	946	6656	0.03
capric acid (C_10:0_)	1106	27,506	0.14
estragole	1163	32,132	0.16
nonanoic acid (C_9:0_)	1198	41,853	0.21
hydocinnamyl alcohol	1200	39,041	0.20
*trans*-2-decenal	1243	44,080	0.22
anethole	1264	95,064	0.48
1,1-dimethoxy decane	1295	20,224	0.10
α-isosafrole	1306	51,940	0.26
ethyl 7-oxooctanoate	1319	51,549	0.26
pimelic acid (C_7:0_)	1380	14,907	0.07
cinnamic acid	1395	595,260	2.99
10-undecenoic acid (C_11:1_)	1397	15651	0.08
azelaaldehydic acid (C_9:0_)	1418	153,561	0.77
suberic acid (C_8:0_)	1436	159,859	0.80
9-hydroxy decanoic acid (C_10:0_)	1448	18,379	0.09
2-tridecanone	1476	11,577	0.06
dimethyl, *p*-phthalate	1489	10,701	0.05
8,8-dimethoxy octanoic acid (C_8:0_)	1495	15,522	0.08
lauric acid (C_12:0_)	1509	80,453	0.40
azelaic acid (C_9:0_)	1519	855,608	4.29
10-oxo-8-decenoate	1546	13,144	0.07
sebacic acid (C_10:0_)	1619	15,469	0.08
myristic acid (C_14:0_)	1705	55,835	0.28
pentanoic acid (C_5:0_)	1875	175,067	0.88
palmitoleic acid (C_16:1_)	1896	19,090	0.10
palmitic acid (C_16:0_)	1907	6,097,000	30.59
heptadecanoic acid (C_17:0_)	2014	16,655	0,08
elaidic acid (C_18:1_)	2056	7,618,000	38.22
9-octadecanoate	2062	1222	0.01
stearic acid (C_18:0_)	2071	2,634,000	13.21
linoleic acid (C_18:2_)	2093	867,137	4.35
7,10,13-eicosatrienoic acid (C_20:0_)	2254	13,237	0.07
arachidic acid (C_20:0_)	2311	12,297	0.06
squalene	2532	13,406	0.07
gluacine	2674	4331	0.02
	total	19,932,647	100.00

Azelaic acid (AZA), a small molecule compound, is known to have several biological functions.^37^ For example, it has antitumor effects on various tumor cells. It promotes the proliferation of NK and T cells and increases the secretion of TNF-α and IFN-γ. Azelaic acid has also been found to increase the expression levels of CD107a and TRAIL in NK cells and CD25 and CD69 in T cells to affect their activation and cytotoxic ability.^37^

Cinnamic acid has been reported to be a highly potent compound for drug development with anti-inflammatory, antioxidant, anticancer, cardiovascular, and antimicrobial effects.^38,39^ As in the case of these previous studies, we believe that cinnamic acid contributes to the biological activities of *H. petaloides* in addition to the other compounds it contains in the present study.

#### Determination of Phenolic Constituents

The phenolic constituents of *H. petaloides* are listed in Table 3; *p*-hydroxybenzoic acid (7.42 μg/g extract), cinnamic acid (6.83 μg/g extract), and gallic acid (5.36 μg/g extract) were the major phenolic compounds. In *Hohenbuehelia serotina*, another species of *Hohenbuehelia*, caffeic acid dimer, epicatechin-3-(3′-*o*-methyl)-gallic acid dimer, quercetin-acetyl-rutinoside, epigallocatechin, rutin, catechin trimer. and 3-caffeoylquinic acid were reported by Wang et al.^40^ Fumaric acid, gallic acid, protocatechuic acid, *p*-hydroxybenzoic acid, and catechin hydrate were detected as phenolic compounds in *Fuscoporia torulosa*, which is another tree mushroom different from *Hohenbuehelia*.^11^ These studies indicate that gallic acid and/or *p*-hydroxybenzoic acid are the mainly available components in other wood fungi as in the case in edible *H. petaloides* of this study.^18,41^

**Table 3:** Phenolic Component Analysis by HPLC-DAD (μg/g Extract)

no	phenolic compounds	RT (min)	*H. petaloides*
1	gallic acid	5.70	5.36
2	protocatechuic acid	8.75	1.83
3	catechin	10.68	
4	pyrocatechol	11.04	
5	chlorogenic acid	12.35	
6	*p*-hydroxybenzoic acid	12.77	7.42
7	6,7-dihydroxy coumarin	14.10	
8	caffeic acid	15.09	
9	3-hydroxybenzoic acid	15.98	
10	syringic acid	16.56	
11	vanillin	17.78	
12	*p*-coumaric acid	20.56	
13	taxifolin	21.26	
14	ferulic acid	22.14	
15	coumarin	24.49	
16	rutin	25.30	
17	ellagic acid	26.11	
18	rosmarinic acid	26.77	
19	myricetin	27.35	
20	quercetin	30.83	
21	cinnamic acid	31.33	6.83
22	luteolin	31.70	
23	hesperetin	32.14	
24	kaempferol	33.21	
25	apigenin	33.77	
26	chrysin	38.40	

*In vitro* and *in vivo* studies have reported that phenolic compounds may play important roles in antioxidant and anti-inflammatory activities. In addition, phenolic compounds exhibit anticancer effects by promoting apoptosis, targeting angiogenesis, and reducing abnormal cell growth.^19,42^ Phenolic compounds are known to be potent antioxidants due to their hydroxyl groups, which play an important role in anticancer, anti-inflammatory, and cardioprotective potential.^42^ Hydroxybenzoic acid and hydroxycinnamic acid are phenolic compounds with high water solubility and bioavailability.^42^ Benzoic and cinnamic acid derivatives have been reported to act as potential candidates for the inhibition of cancer cell proliferation.^43^ The phenolic acid of *p*-hydroxybenzoic acid was reported for antioxidant,^44,45^ anti-inflammatory, and antimicrobial potential.^42^ Gallic acid has been reported to have antioxidant, antimicrobial, anti-inflammatory, and anticancer effects.^46,47^ The results from major phenolic compounds of *H. petaloides* in the present study confirm the relationship between the chemical content and biological activity, as given in the literature.

### Cytotoxicity

The cytotoxic effects of *H. petaloides* mushroom extracts (hexane, methanol, and water) on A549, MCF-7, PC-3, and HT-29 cancer cell lines and healthy human cell line CCD18Co are shown in Table 4. The hexane extract showed cytotoxic activity in all of the cell lines tested. The highest cytotoxic effect (IC_50_ = 26.48 μg/mL) was observed against A549 cells. However, the level of cytotoxicity was low in other cancer cell lines because the IC_50_ values (between 89.89 and 94.66 μg/mL) in other cancer cells were higher than that in the A549 cell line. In addition, the hexane extract exhibited selective cytotoxicity on all cancer cell lines compared with the healthy human cell line CCD18Co (IC_50_ > 200 μg/mL). The hexane extract was dissolved in 10% DMSO. Working dilutions of the extract contained 1% DMSO and did not contribute to cytotoxicity.

**Table 4:** Cytotoxic Effects of *H. petaloides* Extracts (Hexane, Methanol, and Water) on Different Cancer Cell Lines (72 h, Mean ± SD, *n* = 3), *p* < 0.05, NT; Not Tested

	cancer cell lines				
	A549	MCF-7	PC-3	HT-29	human healthy cell line
*H. petaloides* extracts	IC_50_ (μg/mL)				CCD18Co
hexane	26.48 ± 0.02	89.89 ± 0.03	94.66 ± 0.04	93.95 ± 0.02	>200
methanol	138.15 ± 0.06	>200	90.95 ± 0.05	122.15 ± 0.07	>200
water	82.27 ± 0.05	>200	83.18 ± 0.05	135.45 ± 0.06	>200
Reference Materials					
cisplatin	4.28 ± 0.05	NT	NT	2.25 ± 0.06	NT
doxorubucin	NT	1.25 ± 0.02	5.25 ± 0.08	NT	NT

When the cytotoxic activities of other extracts from *H. petaloides* were examined, the methanol extract was found to be the most cytotoxic (IC_50_ = 90.95 ± 0.05 μg/mL) on PC-3 cells in comparison with other cancer cells (Table 4). On the other hand, the water extract exhibited similar cytotoxic effects on both A549 cells (IC_50_ = 82.27 ± 0.05 μg/mL) and PC-3 cells (IC_50_ = 83.18 ± 0.05 μg/mL), which is higher than the cytotoxicity effect (IC_50_ = 135.45 ± 0.06 μg/mL) observed on HT-29 cells. Unlike other extracts, the water extract caused less cytotoxicity (IC_50_ > 200 μg/mL) against the breast cancer cell line MCF-7 and the healthy cell line CCD18Co (Table 4).

The antioxidant, immunomodulatory, hypoglycemic, cytotoxic, and radioprotective activities of polysaccharides from *H. serotina*;^40,48−51^ antimicrobial and antiviral activities of pleurotin isolated from *Hohenbuehelia grisea*;^52^ and antitumor activity of the culture filtrate of *Hohenbuehelia geogenius* ^53^ have been reported in the literature. Wang et al. reported the cytotoxicity effects of *H. serotina* polyphenols on HeLa cells and elucidated the anticancer mechanism.^40^ Sandargo et al. found that the “4-hydroxyipleurogrisein” compound isolated from *H. grisea* showed cytotoxic activity against the murine fibroblast (L929) cell line with IC_50_ = 6.9 μg/mL and against the cervical carcinoma (KB3.1) cell line with IC_50_ = 7.5 μg/mL.^52^ It was reported that *H. serotina* polyphenols inhibited the *in vitro* proliferation of the HeLa cell line at a concentration of 50 μg/mL by 45.04 ± 1.52%.^40^ However, it was reported that *H. serotina* polysaccharides did not show toxicity by in vivo toxicological analysis.^49^ Zhang et al. reported that ribonuclease (RNase) purified from *H. serotina* reduced [3*H*-methyl]-thymidine uptake against leukemia (L1210) and lymphoma (MBL2) cell lines with IC_50_ values of 25 and 40 μM, respectively.^54^ However, the cytotoxic activity has not been studied in other species of *Hohenbuehelia*. The present study indicated that extracts from *H. petaloides* caused cytotoxic effects on different cancer cell lines, and the hexane extract was the most effective. In other words, lipid components from *H. petaloides* were more effective than polyphenols in inducing cytotoxicity on cancer cells.

Murugesan et al. highlighted the importance of fungal lectins because it is important to use naturally resistant proteins to regulate the metastatic stages of cancer.^4^ Numerous naturally occurring carbohydrate-binding proteins are involved in diverse ways in the creation of a novel drug as a curative or preventative intervention for various illnesses. According to *in vitro* findings, *Pleurotus flabellatus* lectin, which belongs to the *Pleurotecae* family, was found to exhibit anticancer activity against colon cancer (HT-29) and lung cancer (A549) cell lines (IC_50_ range: 10–100 μg/mL).^4^ Our next objective is to isolate biologically active extracts that selectively kill cancer cells and support them with *in vivo* studies in order to increase natural bioavailability.

### Effects of Extracts on Apoptosis

#### Investigation of Apoptosis by Flow Cytometry

**Figure 3:**
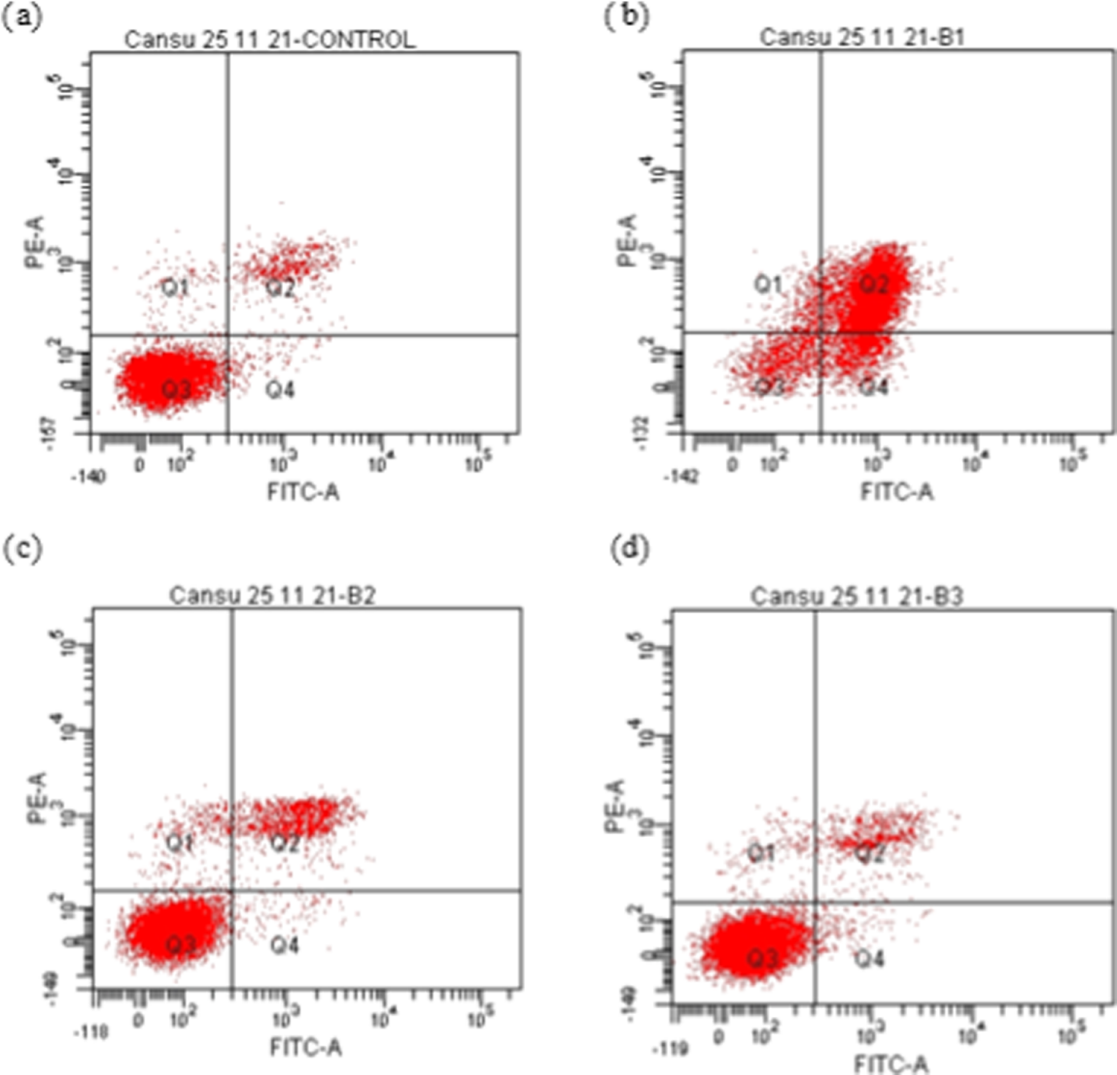
Flow cytometric analysis of A549 cells exposed to different concentrations of the hexane extract from *H. petaloides*: (a) control, (b) 2× IC_50_, (c) IC_50_, and (d) IC_50_ 1/2.

**Figure 4:**
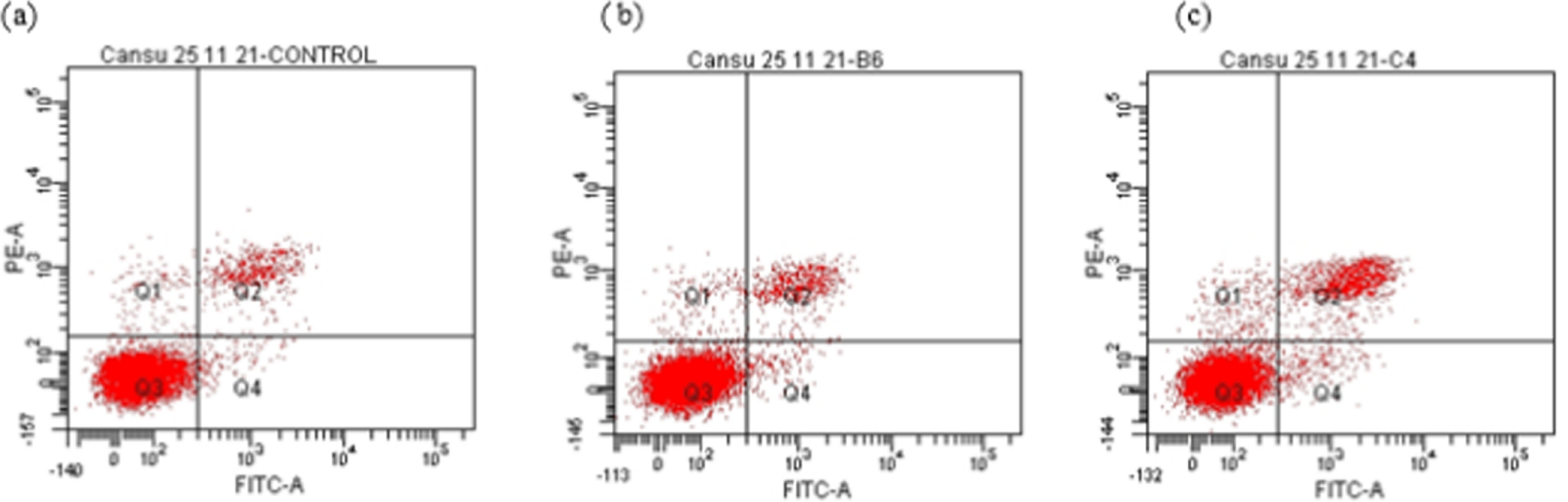
Flow cytometric analysis of A549 cells exposed to different concentrations of the *H. petaloides* water extract: (a) control, (b) IC_50_, and (c) 2× IC_50_.

**Figure 5:**
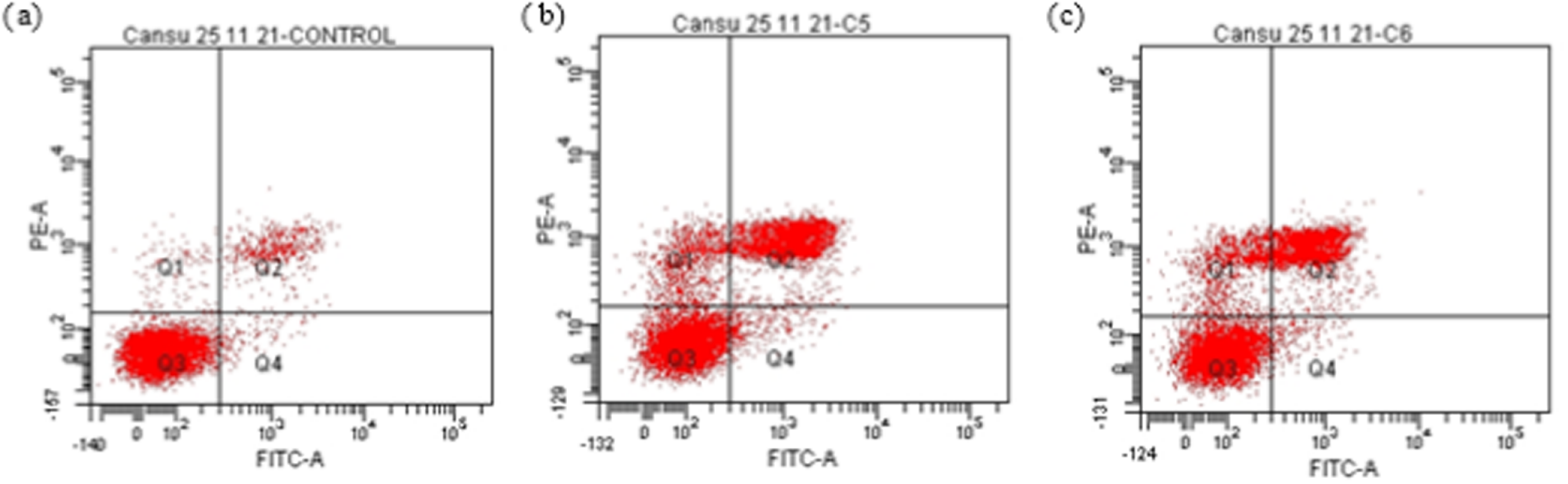
Flow cytometric analysis of A549 cells exposed to different concentrations of *H. petaloides* methanol extract for 24 h: (a) control, (b) IC_50_, and (c) IC_50_1/2.

Cellular cytotoxicity can be induced by different cellular processes. Among them, cell death is the most common one and is classified as apoptosis, necrosis, and autophagy. Apoptosis is programmed cell death, whereas necrosis and autophagy occur when the cells are under stress. In the present study, flow cytometric and reverse transcription polymerase chain reaction (RT-PCR) analyses were carried out to determine if mushroom extracts induce apoptotic cell death. The results indicated that all extracts of *H. petaloides* at different concentrations (IC_50_, IC_50_ 1/2, and 2× IC_50_) induced apoptosis in A549 cells (Figures 3–5 and Table 5). The hexane extract at a 2× IC_50_ concentration caused 74.8% cell death (Table 5). The methanol extract at IC_50_ showed a higher apoptotic effect (32.8%) than the water extract (7.1%).

**Table 5:** Detection of Apoptosis in A549 Cells Incubated with Different Extracts of *H. petaloides* by Flow Cytometric Analyses

	A549 population (%)				
incubation period					
extracts of *H. petaloides*		viable cell (*Q*_3_)	early apoptosis (*Q*_4_)	late apoptosis (*Q*_2_)	necrosis (*Q*_1_)
24 h	2× IC_50_	10.0	11.7	74.8	3.5
	IC_50_	83.9	1.1	12.0	3.0
hexane	IC_50_ 1/2	90.3	1.6	6.4	1.7
24 h	2× IC_50_	83.3	2.5	11.8	2.4
water	IC_50_	89.3	1.8	7.1	1.7
24 h	IC_50_	53.7	2.9	32.8	10.5
methanol	IC_50_ 1/2	57.5	1.9	28.5	12.1
24 h	control	92	1.4	5.4	1.2
untreated					

The hexane extract, which was found to be the most effective extract in terms of cytotoxicity (Table 4), resulted in the highest apoptotic effect compared to the water extract of *H. petaloides*. Even though 74.8% apoptosis was induced with the hexane extract at a 2× IC_50_ value, apoptosis declined to 12% at IC_50_ (Table 5). Among the IC_50_ values of the extracts, methanol was the most effective at inducing apoptosis (32.8%). It was also observed that the level of cytotoxicity (IC_50_ = 26.48 μg/mL; Table 4) correlates with the level of apoptosis (2× IC_50_ = 74.8; Table 5) obtained from the hexane extracts in A549 cells. However, different effects were observed with the methanol and water extracts in terms of cytotoxicity and apoptosis. For example, the water extract was more cytotoxic (IC_50_ = 82.27 μg/mL) than the methanol extract (IC_50_ = 138.15 μg/mL). In contrast, the water extract was less effective on apoptosis (11.8%) than the methanol extract (32.8%). Taken together, the concentrations of water and methanol extracts from *H. petaloides* did not exhibit a linear correlation between cytotoxicity and apoptosis.

Fungal lectins have exhibited a strong affinity for cancer and other related diseases, which is suggestive of their critical involvement in the early and late apoptotic processes that are triggered in response to the inhibition of cellular proliferation.^4^ According to *in vitro* findings, *P. flabellatus* lectin (with IC_50_ values of 67 and 60 μg/mL) significantly reduced apoptosis induction in HT-29 and A549 cell lines.^4^ It has been hypothesized that lectin-induced apoptotic nature causes necrosis, which leads to cell death in cancer cells, and the mushroom-based lectin carrier system4 has been reported as a potential treatment approach to treat this terminal illness.

Zhu et al. reported that palmitic acid inhibited prostate cancer cell proliferation and metastasis by suppressing phosphotidyldinositol 3-kinase/threonine protein kinase, PI3K/Akt pathway.^34^ It has been reported that elaidic acid inhibits human neuroblastoma (SH-SY5Y) cell growth and induces apoptosis by increasing oxidative stress.^36^ We found that the hexane extract tested on the A549 cancer cell line showed a high cytotoxic effect (IC_50_ = 26.48 ± 0.02 μg/mL) compared to other extracts and induced apoptosis in parallel with the increase in concentration. This may be due to the presence of major compounds such as palmitic acid, elaidic acid, linoleic acid, and cinnamic acid in the hexane extract of *H. petaloides*. Further studies are warranted to establish the role of lipid compounds in the control of cell growth in order to find an effective strategy for cancer prevention/treatment.^55^

Many researchers have reported that oxidative stress induced by polyphenols by the accumulation of reactive oxygen species (ROS) by compounds such as gallic acid, ellagic acid, and procyanindin drives cancer cells to apoptosis due to the alteration of the intracellular microenvironment.^45,56^ A polysaccharide from *H. serotina* reduces chromosome abnormalities and micronucleus rates in the bone marrow and blocks the apoptotic pathway of splenocytes in mice exposed to *y*-radiation.^57,58^ In a study conducted by Wang et al., it was found that *H. serotina* polyphenols induced apoptosis in the HeLa cell line, which was reported to be related to the antitumor activity of polyphenols.^40^ In addition to the cytotoxic effect of methanol on the A549 cell line in the present study, the induction of apoptosis (32.8%) by the methanol extract may be most likely related to the phenolic compounds it contains, as indicated in previous studies.

#### Investigation of Apoptosis by Real-Time qPCR Analysis

**Figure 6:**
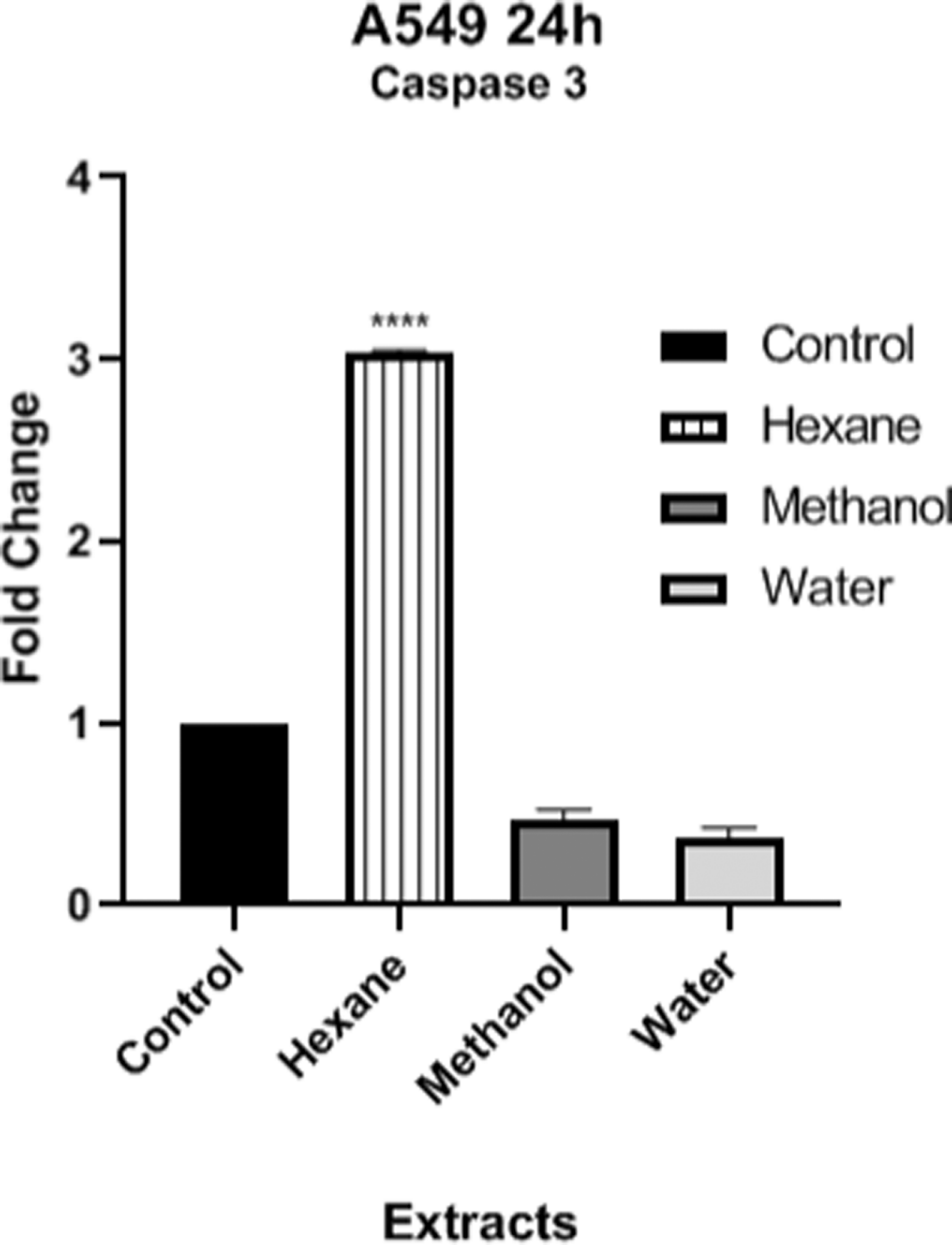
Apoptotic gene *caspase-3* expression in the A549 cancer cell line treated with different extracts of *H. petaloides*.

Caspases are involved in mediating various apoptotic signaling pathways. In the present study, we examined expression profiles of the *caspase-3* gene in A549 cancer cells treated with different extracts of *H. petaolides* at IC_50_ concentrations. The hexane extract at a 2× IC_50_ concentration caused a 3.3-fold increase in caspase-3 expression of the A549 cell line (*p* < 0.05) compared to the control cells (Figure 6). However, methanol and water extracts at the IC_50_ levels resulted in downregulation of the expression of the apoptotic gene *caspase-3* in A549 cells. One reason for this may be the concentration of extracts used in the assay because the hexane extract at 2× IC_50_ concentration caused around 75% cell death in flow cytometry analysis. Another most possible reason is that the phenolic content of the methanol extract may trigger apoptosis through caspase-independent cell death. A number of studies have indicated the occurrence of caspase-independent programmed cell death in different cancer cell lines. For example, resveratrol, a polyphenolic compound, induces caspase-independent apoptosis in mouse prostate adenocarcinoma cells.^59^ Similarly, the low level of caspase-3 expression of A549 cells treated with the methanol extract of *H. petaloides*, rich in phenolic compounds, may indicate the induction of caspase-independent cell death in our present study. Further studies are needed to shed light on the underlying mechanisms. Unlike our results, Wang et al. reported that *H. serotina* polyphenols induced mitochondrial apoptosis pathway in HeLa cells by activation of bax, cytochrome c, and caspase-3.^40^ In addition, *H. serotina* polysaccharides showed radioprotective effect by preventing mitochondrial apoptosis of splenocytes that protect the lysis of erythrocytes in the spleen of mice by inhibition of caspase-3 and caspase 12 gene expression.^49,60^

Apoptotic gene expression in cells was determined by RT-PCR analysis. Statistical analysis of the data was performed using one-way ANOVA; mean ± standard deviation with three replicates is indicated by **** (*p* < 0.0001).

### Anti-Inflammatory Activity

**Figure 7:**
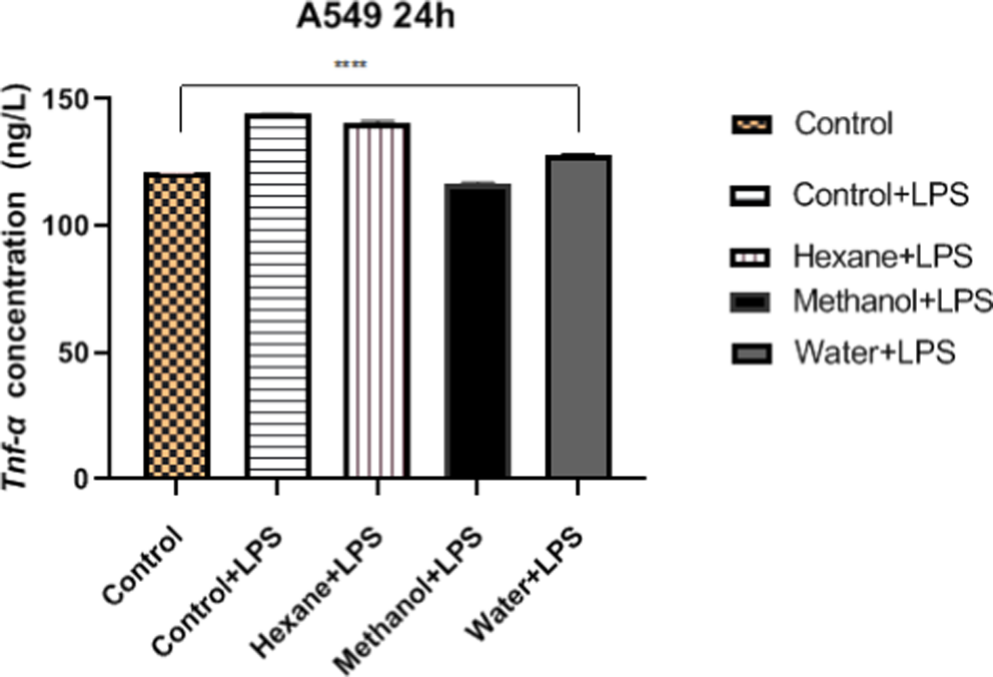
Cytokine (TNF-α) concentration of mushroom extracts in the A549 cell line. Statistical analysis of the data was performed using two-way ANOVA; mean ± standard deviation with three replicates is indicated by **** (*p* < 0.0001).

Cytokines are known to be important mediators of inflammation and cancer progression. In the present study, A549 cells were treated with hexane, methanol, or water extracts of *H. petaloides*, and TNF-α concentrations in the culture supernatants were determined. All extracts reduced TNF-α secretion compared to that in the LPS-induced control (Figure 7). On the basis of TNF-α secretion, the methanol extract was more effective than the hexane and water extracts compared to the control. The methanol extract, in combination with LPS, decreased TNF-α secretion by 2.86-fold compared to the control with LPS (Figure 7). These data showed that the methanol extract of *H. petaloides* can protect A549 cells from the LPS-induced inflammatory response. TNF-α has been reported as one of the inflammatory cytokines that cause tumor formation between inflammation and cancer development.^61^ In the literature, the anti-inflammatory potential of phenolic acids from natural sources has been reported.^42,47^ Similarly, gallic acid and *p*-hydroxybenzoic acid are found to be the major phenolic acids in the methanol extracts of *H. petaloides*. Therefore, the high anti-inflammatory activity of the methanol extract in the present study may be due to the major phenolic compounds, gallic acid and *p*-hydroxybenzoic acid. In addition, Çakır et al. reported that gallic acid showed a cytotoxic effect in A549 and various cancer cell lines and inhibited various signaling pathways involved in cancer formation.^62^

### Angiogenic Activity

**Figure 8:**
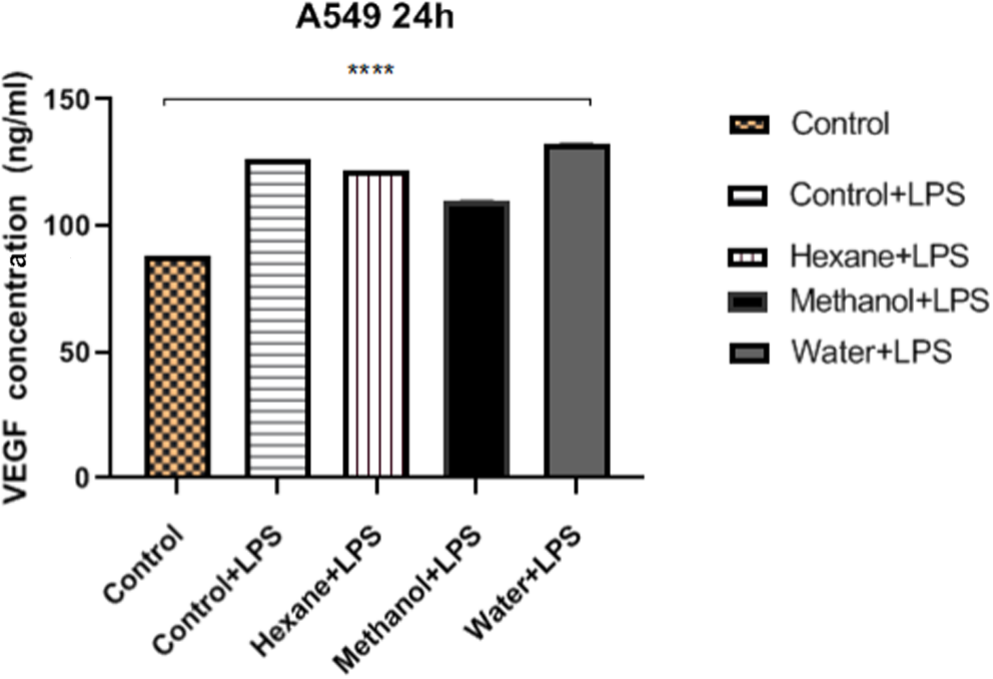
VEGF release from mushroom extracts in the A549 cell line. Statistical analysis of the data was performed using two-way ANOVA; mean ± standard deviation in three replicates is indicated by **** (*p* < 0.0001).

Vascular endothelial growth factor (VEGF) plays a critical role in physiological and pathological angiogenesis.^63^ VEGF is produced by many cell types, including cancer cells. In this study, VEGF secretion was determined after treating A549 cells with hexane, methanol, and water extracts at 200 μg/mL in combination with LPS (10 μg/mL). All extracts with LPS resulted in higher levels of VEGF secretion than the control (Figure 8). Unlike hexane and water extracts, methanol was less effective for VEGF production. However, the water extract caused the highest levels of VEGF secretion compared to other extracts (Figure 8).

Wang et al. reported that palmitic acid inhibits cell invasion by reducing the expression levels of VEGF and CD34 protein.^64^ Similarly, the major lipid-derived compounds palmitic acid (30.59%) and elaidic acid (38.2%) in the hexane extract caused a slight decrease in VEGF secretion compared with the control+LPS in our study. However, the methanol extract was more effective with respect to antiangiogenic activity than the hexane extract. This finding indicates a positive correlation between phenolic compounds and antiangiogenic activity, as shown by Singh and Patra.^65^ In addition, gallic acid was found to be one of the three major phenolic compounds in the methanol extract of *H. petaloides*, and a previous study reported that gallic acid inhibits angiogenesis or metastasis.^68^

In this study, the IC_50_ values of hexane and methanol extracts showed a cytotoxic effect in the lung cancer (A549) cell line, which is in accordance with the fact that they exhibit apoptotic activity in A549 as well as reduce the angiogenic effect compared to the control groups. Our findings that neither extracts show toxicity in healthy human cell lines and reduces the angiogenic effect are promising features against the side effects of chemotherapy drugs in future targeted studies in lung cancer.

### Antioxidant Activity

The antioxidant activities of the *H. petaloides* extracts (hexane, methanol, water) were investigated using BHA, tocopherol, and EDTA standards, and 50% inhibition concentrations were determined (Table 6). The *H. petaloides* methanol extract showed higher activity than the others in all tested methods, and the methanol extract exhibited significant antioxidant activity both in terms of DPPH free radical (IC_50_ = 82.61 ± 0.90 μg/mL) and ABTS cation radical removal (IC_50_ = 55.20 ± 0.65 μg/mL) and CUPRAC-reducing power (IC_50_ = 76.41 ± 0.73 μg/mL). The antioxidant effects of the ethanol extract of *Hohenbuehelia myxotricha* mycelia from different species of *Hohenbuehelia* were investigated.^66^ The TAS, TOS, and OSI values of the ethanol extract of mycelia of *H. myxotricha* were reported as 5.416 ± 0.150, 1.320 ± 0.156, and 0.024 ± 0.003 μmol/L, respectively.^66^ In another study, it was determined that *H. serotina* polysaccharide showed ABTS and hydroxy radical removal activity.^67^ The activity of *H. serotina* against radiation was investigated in irradiated mice, and it was reported that the polysaccharide obtained provided effective protection against radiation-induced damage by increasing antioxidant and immunomodulation activities.^68,69^

**Table 6:** Antioxidant Activity Results of *H. petaloides* Extracts (*p* < 0.05)

mushroom	extract	β-karoten linoleic acid	DPPH	ABTS	metal chelate	CUPRAC
		IC_50_ (μg/mL)				*A*_0.5_ (μg/mL)
H. petaloides	hexane	105.2 ± 0.78	141.8 ± 0.63	101.3 ± 0.80	312.5 ± 1.14	95.67 ± 0.77
	methanol	68.36 ± 0.51	82.61 ± 0.90	55.20 ± 0.65	252.1 ± 1.20	76.41 ± 0.73
	water	136.1 ± 1.05	205.2 ± 0.95	122.8 ± 0.79	>400	127.9 ± 0.96
standards	BHA	1.55 ± 0.05	19.80 ± 0.36	12.63 ± 0.52		25.20 ± 0.05
	α-tocoferol	2.27 ± 0.04	38.50 ± 0.25	34.45 ± 0.35		85.50 ± 0.07
	EDTA				5.50 ± 0.25	

In this study, the methanol extract showed a higher antioxidant effect than the other extracts, and the detected major phenolic compounds, gallic acid,^46,47^*p*-hydroxybenzoic acid,^44,45^ and cinnamic acid^70,71^ were compatible with the literature in terms of antioxidant activity, and we believe that this activity may be due to phenolic compounds. The fact that the methanol extract of the traditionally consumed edible *H. petaloides*, which has high antioxidant activity, showed an anti-inflammatory effect, while it did not show toxicity in a human healthy cell line (CCD18Co) strengthens its potential in the health and industrial fields.

### Antimicrobial Activity

A well diffusion assay was performed to determine if *H. petaloides* extracts had antimicrobial activity. The hexane extract at 2 mg/well concentration resulted in an inhibition zone on the growth of Gram-positive bacteria, *Bacillus cereus* (12.5 mm), *Staphylococcus aureus* (12 mm), *Bacillus subtilis* (14 mm), and *Micrococcus luteus* (15 mm). On the other hand, the methanol extract (9 mg/well) showed antimicrobial activity only against *B. cereus* (14 mm). These findings indicate that the hexane extract has a higher spectrum of antimicrobial activity than that of the methanol extract. This may result from the major lipid components, palmitic acid, elaidic acid, and cinnamic acid, of the hexane extract.^35,38^ Thammasut et al. indicated that short-chain fatty acids exhibit higher antimicrobial activity than long-chain fatty acids.^35^ Therefore, the combination of fatty acids in the hexane extract of *H. petaloides* may contribute to antimicrobial activity in our study.

Similar to our results, Krupodorova et al. indicated that mycelial extracts of *H. myxotricha* exhibited antimicrobial activity against many bacteria such as *Acinetobacter baumannii*, *Enterococcus faecalis*, *Escherichia coli*, *S. aureus*, and *Pseudotricha* sp.^66^

## Conclusions

In this study, the IC_50_ values of hexane and methanol extracts from *H. petaloides* showed cytotoxic effects in the lung cancer A549 cell line, which is in accordance with the fact that they induced apoptosis in A549 cells as well as reduced angiogenic effects. All extracts were selective for cytotoxicity in cancer cell lines compared to healthy cell lines. Higher antimicrobial activity was observed with the hexane extract. We believe that our findings that neither extract showed toxicity in healthy human cell lines and reduced the angiogenic effect are promising for future targeted studies in lung cancer against the side effects of chemotherapeutic products. In order to increase the natural bioavailability, further studies should be carried out for the isolation, structure elucidation, and biological characterization of extracts showing biological activity. which should be confirmed in experimental animals with lung cancer models.
